# Clinical decision rule for cervical magnetic resonance imaging in suspected cervical spinal cord injury without bony injury is useful in predicting severity of cervical stenosis

**DOI:** 10.1186/cc14395

**Published:** 2015-03-16

**Authors:** T Inagaki, A Kimura, A Hagiwara, R Sasaki, K Kobayashi, A Inaka, G Makishi

**Affiliations:** 1 National Center for Global Health and Medicine Hospital, Tokyo, Japan; 2 Seirei Hamamatsu General Hospital, Shizuoka, Japan

## Introduction

Cervical spinal cord injury (CCI) without bony injury (CCIWOBI) is more frequent among Asian than among Caucasian populations and shows various extents of severity. Cervical magnetic resonance imaging (MRI) is useful for detecting intramedullary lesions, ligament injuries and intervertebral disk hernias, but some patients with mild CCIWOBI do not show clinically significant abnormalities on MRI. To date, the cost-benefit ratio of performing MRI in addition to computed tomography (CT) is unclear. We have developed a clinical decision rule for cervical MRI (MR-CDR), indicating MRI for patients >70 years old with ossification of the posterior longitudinal ligament on CT or injury in a ground-level fall or a fall down stairs. The objective of the present study was to prospectively validate this MR-CDR for cervical MRI in patients with suspected mild CCIWOBI.

## Methods

We have been conducting a prospective observational study in two institutions in Japan since September 2012, enrolling patients with CCIWOBI among head or neck trauma patients >16 years old brought in by ambulance. We collect data about patient characteristics, injury profiles, neurological findings, results of radiological examinations, and medical courses. We then analyze the sensitivity and specificity of MR-CDR for detecting intramedullary lesions on MRI and conduct further analysis.

## Results

During the study period, 63 patients were brought in with CCIWOBI. Mean age was 60.6 years (standard deviation, 17.9 years) and 76% were male. Forty-five patients presented with mild symptoms (Frankel Grade D). Cervical MRI was performed for 23 patients. Sensitivity and specificity of MR-CDR in detecting intramedullary lesions on T2weighted imaging among cases of suspected mild CCIWOBI were 85.7% (95% confidence interval (CI), 60.1 to 96.0%) and 33.3% (95% CI, 12.1 to 35.4%). Further analysis showed a significant difference in minimal spinal canal diameter as measured on sagittal T2-weighted imaging between the MR-CDR-positive and MR-CDR-negative groups (5.0 mm vs. 8.3 mm, *P *= 0.0003). One patient underwent surgery during hospitalization and no patients experienced exacerbated neurological findings. No significant differences were evident between groups in discharge status, duration of hospitalization, or neurological findings at discharge.

## Conclusion

MR-CDR was not validated for predicting the existence of intramedullary lesions on cervical MRI. MR-CDR is useful in predicting the severity of cervical stenosis.

